# Quercetin-Conjugated Superparamagnetic Iron Oxide Nanoparticles Protect AlCl_3_-Induced Neurotoxicity in a Rat Model of Alzheimer’s Disease *via* Antioxidant Genes, APP Gene, and miRNA-101

**DOI:** 10.3389/fnins.2020.598617

**Published:** 2021-02-25

**Authors:** Elnaz Amanzadeh Jajin, Abolghasem Esmaeili, Soheila Rahgozar, Maryam Noorbakhshnia

**Affiliations:** ^1^Department of Cell and Molecular Biology and Microbiology, Faculty of Biological Science and Technology, University of Isfahan, Isfahan, Iran; ^2^Department of Plant and Animal Biology, Faculty of Biological Science and Technology, University of Isfahan, Isfahan, Iran

**Keywords:** Alzheimer’s disease, AlCl_3_, quercetin, miR-101, superparamagnetic iron oxide nanoparticle, antioxidant

## Abstract

Alzheimer’s disease (AD) is a neurodegenerative disease with cognitive impairment. Oxidative stress in neurons is considered as a reason for development of AD. Antioxidant agents such as quercetin slow down AD progression, but the usage of this flavonoid has limitations because of its low bioavailability. We hypothesized that quercetin-conjugated superparamagnetic iron oxide nanoparticles (QT-SPIONs) have a better neuroprotective effect on AD than free quercetin and regulates the antioxidant, apoptotic, and APP gene, and miRNA-101. In this study, male Wistar rats were subjected to AlCl_3_, AlCl_3_ + QT, AlCl_3_ + SPION, and AlCl_3_ + QT-SPION for 42 consecutive days. Behavioral tests and qPCR were used to evaluate the efficiency of treatments. Results of behavioral tests revealed that the intensity of cognitive impairment was decelerated at both the middle and end of the treatment period. The effect of QT-SPIONs on learning and memory deficits were closely similar to the control group. The increase in expression levels of APP gene and the decrease in mir101 led to the development of AD symptoms in rats treated with AlCl_3_ while these results were reversed in the AlCl_3_ + QT-SPIONs group. This group showed similar results with the control group. QT-SPION also decreased the expression levels of antioxidant enzymes along with increases in expression levels of anti-apoptotic genes. Accordingly, the antioxidant effect of QT-SPION inhibited progression of cognitive impairment *via* sustaining the balance of antioxidant enzymes in the hippocampus of AD model rats.

## Introduction

Alzheimer’s disease (AD) appears as an outcome of neurodegeneration that is recognized with symptoms of intensive cognitive impairment (Ramalho et al., 2020). AD has a prevalence of ∼45 million people around the world that may rise to ∼150 million by 2050 because of the progressive nature of the disease and limited therapeutic methods. Usually, AD appears in people older than 60 years old (Santamaría et al., 2020). Currently, it has been highlighted that aging is not the only cause of sporadic AD (SAD) development, while environmental and lifestyle factors including malnutrition, air pollution, oxidative stress, etc., play crucial roles in the development and progression of this disease (Steck et al., 2018; Boccardi et al., 2019; Altuna-Azkargorta and Mendioroz-Iriarte, 2020; Cassidy et al., 2020; Jayaraj et al., 2020; Nonaka et al., 2020; Oh and Disterhoft, 2020; Wu et al., 2020). The main AD symptoms include Aβ decomposition and tau hyperphosphorylation (Madav et al., 2019; Zaplatic et al., 2019). During the AD progression, mitochondria impairment leads to increased production of ROS, which, in turn, is the cause of decreased levels of antioxidant enzymes and can result in neural cell death.

On the other hand, the regulation of microRNAs contributes to oxidative stress in the induction of different processes linked to neurodegeneration. Mir101 is known as a key post-transcriptional regulatory element that corresponds to the 3′-untranslated region (3′-UTR) of *App* mRNA, and its overexpression alleviates Aβ production and prevents progression of AD (Lin et al., 2019). In a network analysis study, it was demonstrated that mir-101 is an important regulator of genes related to AD development (Satoh, 2012).

It has been shown that induced ROS production using aluminum chloride (AlCl_3_) in rat models of AD leads to the development of AD-like conditions including the production of Aβ decomposition and oxidative stress (Chavali et al., 2020). Besides, increased activity of inducible nitric oxide synthase (iNOS), the altered expression profile of microRNAs, changes in energy metabolism, and inhibition of serine proteases have been reported as related processes to Al^3+^ exposure. Al^3+^ competes with ferric iron to bind to ROS *via* Fenton dynamics, which, in turn, results in the production of higher levels of oxygen superoxide and ferrous with high redox potential (Bondy, 2014). In this reaction, ROS, Al^3+^, and Fe^2+^ are produced, which all have the potential to enhance oxidative stress within the neural cell (Zatta et al., 2002). Also, it has been found that Al^3+^ can bind to some amino acids of amyloid precursor protein (App) and cause the formation of Aβ sheets (Derry et al., 2020). In the present study, the AD rat model was established *via* oral administration of AlCl_3_ in high dosage (100 mg/kg), which caused behavioral alteration and developed pathological AD-like conditions (Alzahrani et al., 2020; Weng et al., 2020).

Eastern traditional medicine has recommended nature-based treatments to heal several disorders (Alexander et al., 2016). Plant extracts such as ginger, ginseng, curcumin, rutin, and quercetin (QT) are mainly used in traditional medicine as they all have the antioxidant capacity (Farías et al., 2012; Tao et al., 2015; Sreenivasmurthy et al., 2017; Tang and Taghibiglou, 2017). Quercetin is a polyphenol flavonoid that is mainly found in cranberry, red onion, red apple, and green tea (Lozoya-Agullo et al., 2016; Tang et al., 2020). Based on the unique structure of QT, it shows a high antioxidant capacity. QT contains five putative hydroxyl groups on A, B, and C rings. 3′-OH and 4′-OH have been introduced as the most putative groups for molecular interactions (Boots et al., 2008). QT decreases the activity of *iNOS* and increases the activity of superoxide dismutase (SOD) and other antioxidant enzymes by reduction of ROS level in various cell types. Guo and coworkers found that QT and other phenolic compounds could bind to both Fe^2+^ and Fe^3+^ ions (Moura et al., 2015; Tosto and Mayeux, 2017). Meanwhile, QT usage has been limited due to its low bioavailability and low solubility. However, it has been stated that QT is absorbed *via* the intestinal wall while it is converted into its metabolites such as isorhamnetin, tamarixetin, and kaempferol, which have less antioxidant potential. For this reason, various methods have been developed to induce bioavailability and solubility of QT including QT nanoparticles, QT-encapsulated liposomes, and QT encapsulation into PLGA, so that neuroprotective effects of QT encapsulated liposomes have also been demonstrated. Accordingly, our research group decided to develop a nano-sized delivery system and assess its efficiency on different diseases (Najafabadi et al., 2018; Aliakbari et al., 2019; Amanzadeh et al., 2019; Katebi et al., 2019; Yarjanli et al., 2019).

Superparamagnetic iron oxide nanoparticles (SPIONs) have broad applicability in the diagnosis and treatment of different diseases (Musielak et al., 2019; Wilson and Geetha, 2020). SPION not only can distribute through all organs but also can penetrate through BBB and reach brain tissue (Thomsen et al., 2013). In addition, SPION with a negative charge has shown beneficial effects on neurodegeneration at low concentrations (Yarjanli et al., 2017). As mentioned before, long-term usage of QT involving NPs has shown improving effects on cell viability of AD model cell lines. Therefore, the QT-SPION conjugate was proposed as a novel compound, and its antioxidant effects were assessed on cancerous cell lines, learning and memory of healthy rats, and diabetic rat models. In the first step, it was shown that QT-SPIONs were released completely after 8 h using the dialysis method (Najafabadi et al., 2018). In addition, it was shown that the clearance rate of QT-SPION was significantly higher than that of QT, and higher concentrations of QT were observed in plasma and brain tissues of intact rats treated with QT-SPION than those treated with QT, though it showed no significant hepatotoxicity. In the following, it was shown that QT-SPION improves learning and memory in healthy rats while QT did not show considerable effects (Najafabadi et al., 2018). The effect of QT-SPION was also studied on the PC12 cell line treated with H_2_O_2_ as an oxidative agent and demonstrated higher antioxidant potential than QT. In the next step, the QT-SPION effect was studied on diabetes-induced learning and memory impairments, and it was observed that QT-SPION prevented progression of memory impairments more efficiently than QT (Ebrahimpour et al., 2018). According to considerable effects of QT-SPION in different conditions and diseases compared to QT, the authors of the present research decided to study its effects on AD-like symptoms induced by AlCl_3_. Considering that Al^3+^ concentration is significantly high in water pollutions and can cause memory impairment, which is a matter of concern today, on the other side, the usage of AlCl_3_ simulates the gradual progression of memory impairments, which can lead to AD, and AlCl_3_ was used to induce memory impairment to evaluate the effects of QT-SPION.

## Materials and Methods

### Chemicals

Aluminum chloride was purchased from Samchun Co. Quercetin was obtained from Sigma-Aldrich (St. Louis, MO, United States).

### QT-SPION Preparation

We synthesized dextran SPION using a co-precipitation technique as previously reported (Najafabadi et al., 2018). In brief, FeCl_2_ anhydrous [Catalog Number (Cat No). 372870], FeCl_3_ anhydrous (Cat No. 451649), and dextran (Cat No. 1179708) were dissolved in deionized (DI) water, and all were mixed. In the following, the mixture was poured into a three-neck flask equipped with a mechanical stirrer. Then, the pH of the solution reached ∼9 by adding an ammonia solution into the mixture. The solution was kept at 90°C for 2 h with continuous stirring, and then the consequential precipitate was collected using a strong external magnet. The supernatant was washed several times with DI water and ethanol and then was dried in an oven at 70°C overnight. In the following, quercetin (Cat No. Q4951) was added to dextran-coated SPIONs. Fourier transform infrared (FTIR) spectroscopy was used on the Jasco 6300 spectrophotometer (JASCO, Baltimore–Washington, WA, United States) in the transmission model with Kbr pellets in which wave numbers ranged from 400 to 4,000 cm^–1^ to check out conjugation. In addition, an X-ray diffraction (XRD) test was used to evaluate magnetite NPs through Cu Kal (*k* = 1.54056 Å) radiation on a PANalytical XPERT PRO powder XRD at room temperature. The field emission scanning electron microscope (SEM) Hitachi S-4700 equipped with an energy dispersive X-ray analysis detector was used to evaluate the morphological features of synthesized NPs. Also, the molecular weight of SPIONs was 231.533 g/mol.

### Animals

Forty-eight male Wistar rats (weighing 180 ± 20 g, 7 weeks old) were purchased from the animal laboratory of the Physiology Department of the University of Tehran (Tehran, Iran). All procedures were conducted under the guidelines for the care and use of laboratory animals (United States National Institute of Health Publication No 80-23, revised 1996) and were reviewed and approved by the animal ethics committee of the University of Isfahan (Ethics number: IR.UI.REC.1396.065). Four rats were kept in each cage in the animal laboratory for 2 weeks to adapt to the environment. They had free access to food and water and were kept under 12-h light/12-h dark conditions at 24°C set in the lab. Cage beds were covered with shredded wood, which was cleaned and refreshed every 2 days. Then, rats were divided into six groups randomly, with eight rats per group. Groups were categorized as follows:

Group 1: The control group received no treatment.

Group 2: The sham group received 1 ml of distilled water per day.

Group 3: The AlCl_3_ group received AlCl_3_ at 100 mg/kg/day concentration dissolved in 1 ml of distilled water.

Group 4: The AlCl_3_ + SPION group received AlCl_3_ at 100 mg/kg/day and SPION at 25 mg/kg/day concentrations, all dissolved in 1 ml of distilled water.

Group 5: The AlCl_3_ + QT group received AlCl_3_ at 100 mg/kg/day and QT at 25 mg/kg/day concentrations, all dissolved in 1 ml of distilled water.

Group 6: The AlCl_3_ + SPION-QT group received AlCl_3_ at 100 mg/kg/day and QT-SPION at 25 mg/kg/day concentrations, all dissolved in 1 ml of distilled water.

All administrations were performed *via* oral administration (gavage) for 42 continuous days.

### Organs and Body Weight Measurements

Body weight was measured on the 1st, 14th, 28th, and 42nd day of the treatment period, and the weight gain trend was similar in all groups. At the end of the study, based on Anesthesia (Guideline) of Vertebrate Animal Research protocols, animals were sacrificed using a combination of xylazine and ketamine overdose (100 and 10 mg/kg, respectively). Then, the weight of the liver and brain tissues of all animals was measured.

### Behavioral Tests

#### Morris Water Maze

Spatial learning and memory changes were assessed using Morris water maze (MWM). Rats were trained for 5 days as the acquisition phase between the 17th and 20th day of treatment. A black pool with a depth of 50 cm and a diameter of 180 cm was used for this test. The pool was presumably divided into four quadrants including NE (northeast) as quadrant no. 1, SE (southeast) as quadrant no. 2, SW (southwest) as quadrant no. 3, and NW (northwest) as quadrant no. 4. Water depth was 40 cm and an invisible circular platform with a diameter of 10 cm was placed in quadrant no. 3. The platform was placed 1 cm below the water level, and water was made opaque using powdered milk. Three signs were installed on the walls around the pool in the dark experiment room so that rats could use them to assign the paths toward the platform. A video camera was set to the ceiling on the top of the pool with a computerized tracking system to track all movements of rats with details including path, speed, and duration (VideoTracking Software, designed by BorjSanat Company). Each rat was trained for four rounds every day, and in each training round, they were released from a different quadrant while all the time platform was at quadrant no. 3. Swimming path, swimming speed, and escape latency (latency to find the platform) were recorded using a video tracking system that was set on the top of the black pool. The 1-h gap was considered between every training round for each rat.

Probe trials were performed twice on the 21st and 42nd day of treatment. For this test, the invisible platform was removed. Each rat was released into the water gently and they were allowed to swim for 60 s. In these tests, time spent in the target quadrant in which platform was placed at training days (time spent in target zone), time spent in the opposite quadrant, and the number of crosses (plate crosses) on the platform were recorded.

#### Passive Avoidance Test

The passive avoidance test was used to assess fear-induced learning and memory changes in studied rats. The shuttle box apparatus is composed of two compartments including light and dark, each with dimensions of 30 cm × 20 cm × 25 cm, which is separated *via* a guillotine door. Similar to MWM, in this test, training was performed once on the 19th day of treatment. The test strategy is based on the desire of rats to stay in a dark place. Animals were placed in the light compartment when the door was closed and kept there for 5 s. Then, the door was opened and rats were allowed to move and pass to the dark compartment. As soon as the whole body of rats moved into the dark compartment, the door was closed and an electric shock was applied as 0.5 mA for 1 s. Rats were allowed to stay in the dark compartment for 30 s and then were transferred to their home cages. After 1 h, they were put in the light compartment and this turn took 300 s. If rats moved to a dark compartment sooner than 120 s, they received another shock. A maximum of five training rounds were considered, and if any rat did not learn, it was removed from the test.

**FIGURE 1:**
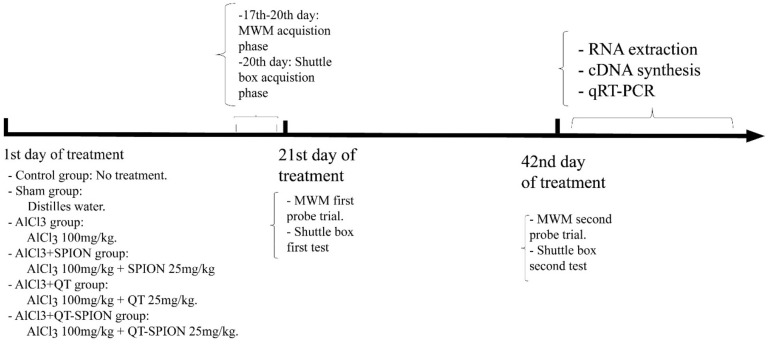
Timeline of experiments. MWM, Morris water maze; qRT-PCR, quantitative real-time polymerase chain reaction.

The first trial was performed 24 h later while the guillotine door was removed. A duration of 120 s was considered for the test. Any rat that spent less time in the dark compartment and moved later in the dark compartment showed better avoidance memory. The second trial was performed on the 42nd day of treatment in order to evaluate the effect of AlCl_3_ and treatments. The timeline of behavioral tests is presented in Figure 1.

### Acetylcholine Esterase (AChE) Activity Assay

AChE activity is elevated in the brain tissue of patients with AD. Accordingly, its activity is measured as a confirmation for developing the AD model and to show the effect of treatment. In this study, Ellman’s method was applied (Oikarinen et al., 1983; Ademosun et al., 2016; Song et al., 2020). Hippocampus tissues were dissolved in 0.25 M sucrose buffer and were maintained for 30 min. In the following, samples were centrifuged at 9,500 rpm, and the supernatant was used to assess Acetylcholine concentration using spectrophotometry. Absorption was read at 412 nm and results are presented as μmol/h/mg.

### Metal Ion Concentrations in the Hippocampus

Twenty-five milligrams of the hippocampus of sacrificed animals was put in 3 ml of acid nitric (HNO_3_ and 65%) and maintained for 18 h. In the following, the prepared solution was filtered using filter paper and the clear solution was obtained and assessed *via* inductively coupled plasma mass spectrometry (ICP-MS) manufactured by Perkin Elmer Company as Optima 8300 ICP-OES model.

### Molecular Studies

#### mRNA Quantitative Assessment

One hundred milligrams of hippocampus tissue was weighed, and total RNA of samples was extracted using TRIzol *via* an ethanol-based method (Cat No. 15596026, Invitrogen). The mixture was merged *via* pipetting 50–60 times using two different needles (28 G 1/2 and 27 G 1/2). Then, it was centrifuged at 12,000 × *g* for 5 min at 4°C due to the high-fat content of hippocampus tissue. After 5 min, 0.2 ml of chloroform was added and mixed using a vortex, and samples were centrifuged at 12,000 × *g* at 4°C for 15 min. The supernatant was transferred into a new tube and 0.5 ml of isopropanol was added to it. The mixture was centrifuged for 10 min at 4°C at 12,000 × *g*, and the supernatant was removed. In the last step, RNA sediment was washed using 1 ml of ethanol and centrifuged at 7,500 × *g* at 4°C for 10 min. The concentration of extracted RNA was measured using the NanoDrop spectrophotometer (NanoDrop Spectrophotometer manufactured by Thermo Scientific) at a 260/280 rate. DNase1 treatment was applied using RNase free DNAse1 from the Fermentase company (Cat No. MAN0012000). RNA was converted into cDNA using the PrimeScript RT Reagent Kit (Perfect Real Time) with a Catalog Number of 15596026 (Takara Company, Cat No. rr037Q). Obtained cDNA was also checked using the NanoDrop spectrophotometer. Primers were designed using Allele ID software version 7.84 (PRIMER Biosoft) as can be observed in Table 1. *T*_*m*_ of all designed primers was set at 58°C. Quantitative real-time PCR was performed using RealQ Plus Master Mix Green (from Ampliqon Company and Cat No: A323402). Messenger RNA expression of *GPX1*, *iNOS1*, *SOD1*, *APP*, *BAX*, and *BCL2* was evaluated using quantitative real-time PCR (qRT-PCR), and samples were normalized to β-Actin as a housekeeping gene. The PCR primers that were validated experimentally across rat transcriptome were used in the qRT-PCR experiment. qRT-PCR was performed using the Bio-Rad Chromo4^TM^ detector (Cat No. CFB-3240). All the experiments were performed in duplicate with polymerase activation at 95°C for 30 s, cDNA denaturation at 95°C for 15 s, annealing, and extension at 60°C for 30 s including 35 cycles. The melting curve analysis was performed at 65–95°C intervals per reading step. The fold change values were calculated with the comparative Ct method (2^–ΔΔ*Ct*^).

**TABLE 1:** Primers designed for the qRT-PCR experiment.

Gene	Sense (5′-3′)	Antisense (3′-5′)	GenBank ID of cDNA
β-Actin	CTCTATGCCAACACAGTG	AGGAGGAGCAATGATCTT	AF541940.1
*APP*	TACTGCCAAGAGGTCTAC	CGGTAAGGAATCACGATG	BC062082.1
iNOS	TTAAGGAAGTAGCCAATGC	TCAGAGCCATACAGGATAG	NM_012611.3
*SOD1*	CACGAGAAACAAGATGACT	AGACTCAGACCACATAGG	BC082800.1
*BCL2*	GTGGATGACTGAGTACCT	GCCAGGAGAAATCAAACA	L14680.1
*BAX*	TTTGCTACAGGGTTTCATC	ATGTTGTTGTCCAGTTCAT	U32098.1
*GPX-1*	AGTTCGGACATCAGGAGAATG	TCACCATTCACCTCGCACT	NM_030826.4
*CAT*	TAAGACTGACCAGGGCATC	CAAACCTTGGTGAGATCGAA	AH004967.2

#### MicroRNA Quantitative Assessment

Total RNA with a concentration of 1 μg/μl was used to polyadenylate all microRNA content using the BON-miR qPCR kit (manufactured by Stem cells tech company, Iran. Cat No. BN-0011.17). Two microliters of total RNA was mixed with rATP (10 mM), 10 × po*ly-A* polymerase buffer, and po*ly-A* polymerase, and was set up to 10 μl using RNase-free water. The mixture was incubated at 37°C for 30 min, and immediately after that, 65°C was applied for 20 min, which aimed to inactivate po*ly-A* polymerase.

In order to add the adapter, poly-A microRNAs were mixed with the BON-RT adapter and the solution was incubated at 75°C for 5 min. RT enzyme, dNTP, 5′-RT buffer, and RNase-free water were added to the mixture up to 20 μl. This mixture was incubated in a serial thermal condition including 25°C for 10 min, 42°C for 40 min, and 70°C for 10 min.

In the following, RT-PCR was performed using obtained cDNA, miRNA-specific forward primer, universal reverse primer, 2 × miRNA QPCR master mix, and nuclease-free water and set up to 13 μl. Finally, the RT-PCR program was set as 1 cycle including 2 min at 95°C and 40 cycles including 95°C for 5 s and 60°C for 30 s.

### Gene Ontology Analysis

Gene Ontology (GO) analysis was performed to confirm the roles of studied genes in oxidative stress and cell death. For this purpose, we used the BINGO application through the Cytoscape platform to find the functional pathways in which all studied genes are associated with (Shannon et al., 2003).

### Statistical Analysis

The obtained results were compared using GraphPad Prism V_8__.0_ for Windows. Parameters measured in behavioral tests were assessed *via* two-way ANOVA and then Tukey’s multiple comparison test while significance level was considered for comparison with and between the groups. ICP, AChE activity, and expression levels of genes were assessed *via* one-way ANOVA and then Dunnett’s multiple comparison test. All data are represented as mean ± standard error of the mean (SEM) and *p* < 0.05 was considered as statistically significant.

## Results

### SPION Characterization

**FIGURE 2:**
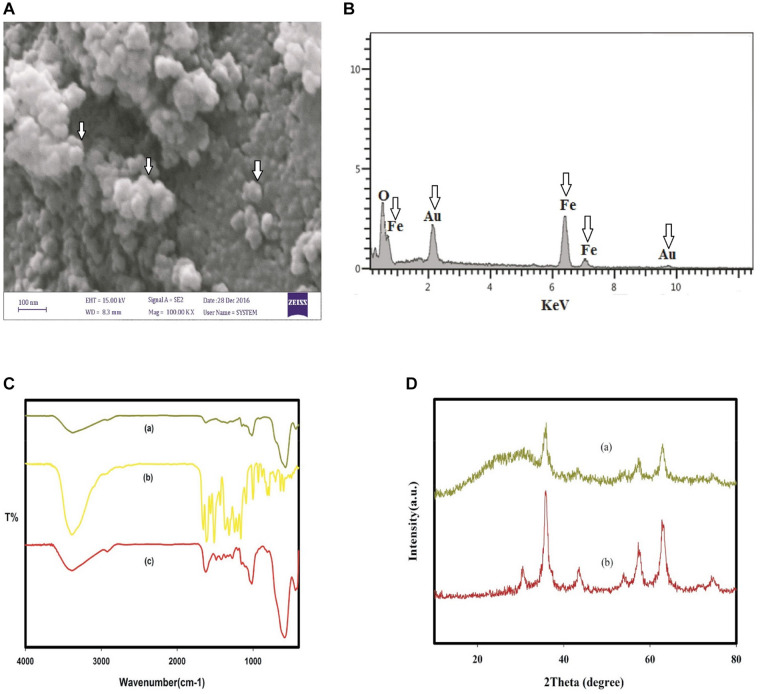
Characterization of QT-SPION. **(A)** Scanning electron microscopy (SEM) image for QT-SPION. **(B)** SEM-EDX spectrum for QT-SPION. Arrows show the existence of Fe in nanocomposition. **(C)** FTIR spectra of dextran-coated SPION (a), QT (b), and QT-SPION (c). **(D)** XRD pattern obtained for dextran-coated SPION (a) and QT-SPION (b). QT-SPION, quercetin-superparamagnetic conjugate; FTIR, Fourier transform infrared; XRD: X-ray diffraction.

Assessment of physical and chemical characteristics of QT-SPION conjugates using FTIR spectroscopy showed a strong band at 3,386 cm^–1^, which belongs to the vibrations in hydroxyl groups and also the confirming bands of the conjugation of QT to SPIONs. In addition, the XRD pattern of obtained QT-SPION demonstrated similarity to the pattern of crystalline magnetite SPION. Results of scanning electron microscopy revealed QT-SPION conjugates with spherical shapes with a size range of 30–50 nm. The energy-dispersive X-ray detector (EDX) results confirmed the presence of iron and oxygen elements in the synthesized particles (Figure 2).

### Effect of Treatments on Body and Organs Weight

**FIGURE 3:**
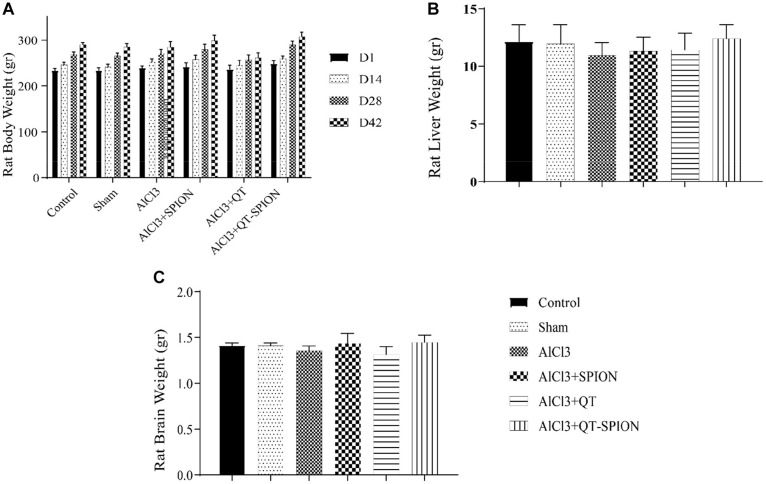
Results of one-way ANOVA for **(A)** body weight of rats in six groups at 1, 14, 28, and 42 days of treatment. **(B)** Liver weight of rats after sacrifice on the 42nd day. **(C)** Brain weight of rats after sacrifice on the 42nd day. Data are presented as mean ± SEM (*n* = 8); ^∗∗∗^*p* < 0.001, ^∗∗^*p* < 0.01, and ^∗^*p* < 0.05 are statistically different in comparison with all groups. ANOVA, analysis of variance.

Figure 3A shows the results of body weight comparison of rats on the 1st, 14th, 28th, and 42nd day of treatment using the two-way ANOVA test. There was no significant difference between treatment groups and the control group considering *p* < 0.05. Results of one-way ANOVA revealed no significant difference between the weight of animals’ liver (Figure 3B) and brain (Figure 3C) (*p* < 0.05).

### Effects of Treatments on Behavioral Performance

#### MWM

**FIGURE 4:**
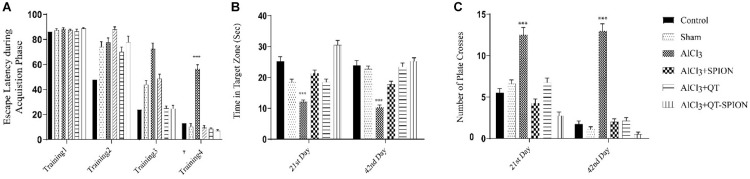
Effect of QT-SPION on rats treated with AlCl_3_ simultaneously in the MWM test. Test results are presented as mean ± SEM (*n* = 8; *p* < 0.001): **(A)** The escape latency during the acquisition phase including four training days of treatment (17th, 18th, 19th, and 20th day of treatment): a significant increase in AlCl_3_ group in comparison with control and AlCl3 + QT-SPION groups. **(B)** The time spent in the target zone at both probe trial days (21st and 42nd): shorter time spent by the AlCl_3_ group compared to control and AlCl3 + QT-SPION groups. **(C)** The number of plate crosses in the AlCl_3_ group is significantly more than that in the control group. Data are presented as mean ± SEM (*n* = 8); ^∗∗∗^*p* < 0.001, ^∗∗^*p* < 0.01, and ^∗^*p* < 0.05 are statistically different in comparison with all groups. SEM, standard error of mean; MWM, Morris water maze.

The MWM test revealed that aluminum chloride treatment causes a significantly larger escape latency of AlCl_3_ compared to the control group. This result shows the destructive effect of AlCl_3_ on the cognitive functions of rats. Alternatively, treatment with QT-SPION confronted an increase in escape latency caused by AlCl_3_ on both the 21st and the 42nd day (*p* < 0.001). In addition, the AlCl_3_ + QT-SPION group demonstrated smaller escape latency with AlCl_3_ + SPION and AlCl_3_ + QT groups and improved spatial memory (Figure 4).

#### Passive Avoidance Test

**FIGURE 5:**
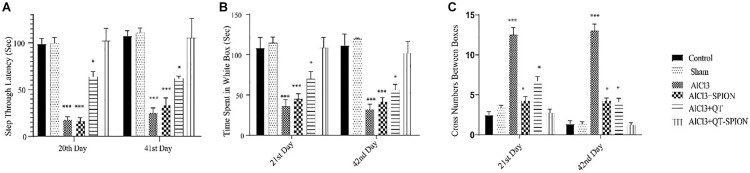
Effect of QT-SPION treatment on step-through latency of rats using passive avoidance in AlCl_3_-treated groups. Data are presented as mean ± SEM (*n* = 8); ^∗∗∗^*p* < 0.001 in comparison with all groups. **(A)** Step-through latency on the training day. **(B)** Time spent in the white box on days 21 and 42. **(C)** Retention latency *via* cross number between boxes on days 21 and 42. AlCl_3_, aluminum chloride; QT-SPION, quercetin-superparamagnetic iron oxide nanoparticles; SEM, standard error of mean.

In this test, the time that each rat should pass from the white box to the black box, called step-through latency (STL), was recorded on the 20th day of treatment, while retention latency, the time that the rat stays in the white box on the trial day, was recorded on the 21st and 42nd day of treatment. A meaningful decrease was observed in RL in the AlCl_3_ group in comparison with the control group (*p* < 0.001), which shows the impaired spatial learning of rats caused by AlCl_3_. However, QT-SPION treatment simultaneously with AlCl_3_ treatment in the AlCl_3_ + QT-SPION group led to similar results with the control group on the 21st and 42nd day, which reveals the recovery effect of QT-SPION and the memory impairment effect of AlCl_3_ treatment. The AlCl_3_ + QT and AlCl_3_ + SPION groups showed significantly higher STL and RL on the 21st and 42nd day of treatment in comparison with AlCl_3_, while improvements in STL and RL in these groups were lower than the control, sham, and AlCl_3_ + QT-SPION groups (*p* < 0.01) (Figure 5).

#### AChE Activity

**FIGURE 6:**
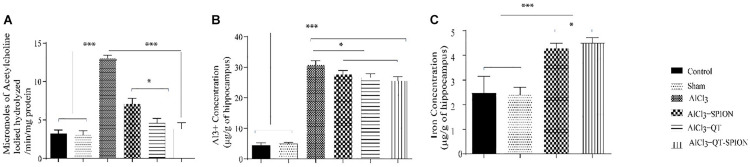
**(A)** Effect of QT-SPION treatment on acetylcholinesterase activity in the hippocampus of AlCl_3_-treated rats with induced oxidative stress. **(B)** Concentration of Al^3+^ ions in the hippocampus of rats in AlCl_3_, AlCl_3_ + SPION, AlCl_3_ + QT, and AlCl_3_ + QT-SPION groups. **(C)** The concentration of iron in the hippocampus of rats in AlCl_3_ + SPION and AlCl_3_ + QT-SPION groups. Data are presented as mean ± SEM (*n* = 8); ^∗∗∗^*p* < 0.001, ^∗∗^*p* < 0.01, and ^∗^*p* < 0.05 are statistically different in comparison with all groups. QT-SPION, quercetin-superparamagnetic iron oxide nanoparticle; AlCl_3_, aluminum chloride; SEM, standard error of mean; ICP, inductively coupled plasma mass spectroscopy.

Treatment with aluminum chloride showed a significant increase in activity of the AChE enzyme (*p* < 0.001) in hippocampal neurons of rats in the AlCl_3_ group compared to the control and sham groups. On the other hand, the adverse effect caused by AlCl_3_ was reversed in the AlCl_3_ + QT and AlCl_3_ + QT-SPION groups; however, results obtained for the AlCl_3_ + SPION group showed no significant difference with the AlCl_3_ group (Figure 6A).

### Metal Ion Concentrations

Since we used aluminum chloride to develop the AD model, we measured the accumulation of this ion in the brain of rats. Results of ICP for aluminum ion in brain tissue demonstrated that all treatments had significantly higher Al^3+^ content in comparison with the control and sham groups (*p* < 0.001) (Figure 6B). The iron content of the AlCl_3_ + SPION and AlCl_3_ + QT-SPION groups was significantly higher than that of the other groups (*p* < 0.001), and the iron content of AlCl_3_ + SPION is also higher than that of the AlCl_3_ + QT-SPION group (Figure 6C).

### qRT-PCR

**FIGURE 7:**
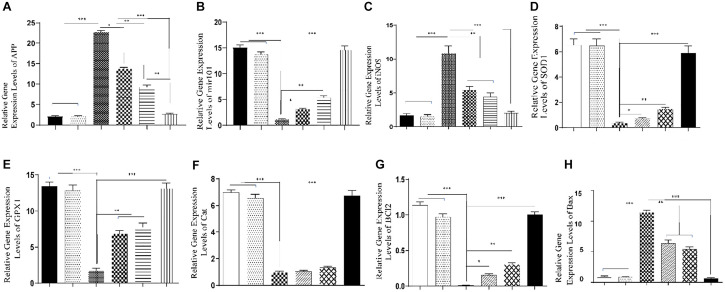
Effect of QT-SPION treatment on relative gene expression levels in the hippocampus of rats treated with AlCl_3_. **(A)** A significant decrease in expression levels of *APP* in the hippocampus of AlCl_3_ + QT-SPION group rats in comparison with the AlCl_3_ group. **(B)** A significant increase in expression levels of *mir101* in the hippocampus of AlCl_3_ + QT-SPION group rats in comparison with the AlCl_3_ group. **(C)** A significant decrease in expression levels of *iNOS* in the hippocampus of AlCl_3_ + QT-SPION group rats in comparison with the AlCl_3_ group. **(D)** A significant increase in expression levels of *SOD1* in the hippocampus of AlCl_3_ + QT-SPION group rats in comparison with the AlCl_3_ group. **(E)** A significant increase in expression levels of *GPX1* in the hippocampus of AlCl_3_ + QT-SPION group rats in comparison with the AlCl_3_ group. **(F)** A significant increase in expression levels of *CAT* in the hippocampus of AlCl_3_ + QT-SPION group rats in comparison with the AlCl_3_ group. **(G)** A significant increase in expression levels of *BCL2* in the hippocampus of AlCl_3_ + QT-SPION group rats in comparison with the AlCl_3_ group. **(H)** A significant increase in expression levels of *BAX* in the hippocampus of AlCl_3_ + QT-SPION group rats in comparison with the AlCl_3_ group. Data are expressed as mean ± SEM (*n* = 8); ^∗∗∗^*p* < 0.001, ^∗∗^*p* < 0.01, and ^∗^*p* < 0.05 are statistically different in comparison with all groups. QT-SPION, quercetin-superparamagnetic iron oxide nanoparticle; AlCl_3_, aluminum chloride; SEM, standard error of mean; *APP*, amyloid precursor protein; *mir101*, microRNA 101; *iNOS*, inducible nitric oxide synthase; *SOD1*, superoxide dismutase 1; *GPX1*, glutathione peroxidase 1; *CAT*, catalase.

In the AD development process, elevated *App* expression level is a key factor. Results of qRT-PCR showed that transcription levels of the *App* gene significantly increased in the hippocampus of rats in the AlCl_3_ group compared to the control and sham groups, while the difference between AlCl_3_ and the AlCl_3_ + SPION, AlCl_3_ + QT, and AlCl_3_ + QT-SPION groups was statistically significant; however, the AlCl_3_ + QT-SPION group showed similar results to the control (*p* < 0.001). Interestingly, the difference between AlCl_3_ + QT and AlCl_3_ + QT-SPION was also significant, which means that levels of *App* transcript of the AlCl_3_ + QT-SPION group are significantly lower than AlCl_3_ + QT, while it is more similar to the control group than any of the treatment groups (Figure 7A).

In the AD development process, decreased *mir-101* expression levels enhance *App* expression. Results of qRT-PCR for *mir-101* showed that the AlCl_3_ group has a significantly lower expression level for this microRNA compared to the control group, while AlCl_3_ + SPION revealed significantly lower expression levels compared to the control and sham groups. On the other hand, expression levels of *mir-101* were significantly higher than those of the AlCl_3_ group. Besides, the AlCl_3_ + QT and AlCl_3_ + QT-SPION groups showed drastically higher expression levels compared to the AlCl_3_ group (*p* < 0.001) (Figure 7B).

Oxidative stress plays a key role in AD progression and increased *iNOS* expression level is a key factor in enhancement of oxidative stress. Transcription levels of the *iNOS* gene significantly increased in the AlCl_3_ group compared to the control group, while treatment with QT-SPION prevented increase in *iNOS* expression levels in the AlCl_3_ + QT-SPION group compared to the AlCl_3_ group (*p* < 0.05). In addition, SPION and QT treatments reduced expression levels of *iNOS* in comparison with the AlCl_3_ group (Figure 7C). Transcription levels of the *SOD1* gene significantly decreased in the AlCl_3_ group compared to the control group, but simultaneous treatment with QT-SPION compensated the effect of AlCl_3_ in the AlCl_3_ + QT-SPION group compared to the AlCl_3_ group (*p* < 0.001). Besides, the results obtained for the AlCl_3_ + QT and AlCl_3_ + SPION groups were significantly higher than those for the AlCl_3_ group (*p* < 0.001) (Figure 7D).

In the AD progression process, expression levels of antioxidant enzymes including *GPX1* and *CAT* and apoptotic genes such as *BCL2* and *BAX* decrease. Results of qRT-PCR revealed that transcription levels of the *GPX1* gene decreased drastically in AlCl_3_ compared to the control group (*p* < 0.001). However, meaningful increases were observed in the AlCl_3_ + QT-SPION group compared to the AlCl_3_ group, which is a similar state to the control group. A significant increase was observed in the AlCl_3_ + SPION and AlCl_3_ + QT groups in comparison with the AlCl_3_ group (*p* < 0.01), while the results obtained for these groups were still significantly lower than those for the control group (Figure 7E).

Results of qRT-PCR for the *CAT* gene revealed meaningful lower expression levels in the AlCl_3_ group compared to the control (*p* < 0.001). On the other hand, the AlCl_3_ + QT-SPION group showed significantly higher expression levels than the AlCl_3_ group and results obtained for AlCl_3_ + QT-SPION was similar to the control group. Noteworthy, the AlCl_3_ + QT and AlCl_3_ + SPION groups revealed no significant increase in expression levels of *CAT* gene compared to the AlCl_3_ group (Figure 7F).

qRT-PCR results revealed that expression levels of *BCL2* as an antiapoptotic gene showed a significant decrease in the AlCl_3_ group compared to the control group (*p* < 0.001); however, simultaneous treatment of AlCl_3_ and QT-SPION in the AlCl_3_ + QT-SPION group led to a recovery effect against the destructive effect of AlCl_3_ compared to the AlCl_3_ group (Figure 7F). Results of qRT-PCR for *BAX* as a proapoptotic gene showed significantly higher expression levels in the AlCl_3_ group compared to the control group (*p* < 0.001), while this effect was recovered in the AlCl_3_ + QT-SPION group *via* treatment with QT-SPION, and these results were similar to the control group (Figure 7H).

### GO Analysis

Based on the results obtained from BINGO, it was found that regulation of programmed cell death, response to inorganic substances, neuron cell death regulation, and response to ROS were the most frequent functions related to memory impairment and AD-like conditions, which are induced by AlCl_3_ treatment and mostly are recovered by QT-SPION treatment. All the related information is listed in Table 2.

**TABLE 2:** Results of GO analysis: the list of the most frequent regulatory pathways in which studied genes are involved.

GO ID	GO description	*P* value	Cluster frequency	Genes
42,981	Regulation of apoptosis	2.3444E-8	85.70%	*APP, GPX1, CAT, BCL2, BAX, and SOD1*
43,067	Regulation of programmed cell death	2.5246E-8	85.70%	*APP, GPX1, CAT, BCL2, BAX, and SOD1*
43,066	Negative regulation of apoptosis	3.2890E-	71.40%	*GPX1, CAT, BCL2, BAX, and SOD1*
10,035	Response to inorganic substance	3.5499E-8	71.40%	*GPX1, CAT, BCL2, BAX, and SOD1*
43,523	Regulation of neuron apoptosis	3.7724E-8	71.40%	*GPX1,CAT,BCL2,BAX, and SOD1*
42,743	Hydrogen peroxide metabolic process	4.3813E-8	42.80%	*GPX1, CAT, and SOD1*
48,518	Positive regulation of the biological process	1.6180E-7	100%	*APP, GPX1, CAT, BCL2, BAX, INOS, and SOD1*

## Discussion

Air and water pollutants can specifically influence brain tissue and cause memory impairment, which, in turn, can lead to AD or other neurodegenerative disorders. Among the heavy metals, aluminum has shown harmful effects on memory and learning *via* induction of oxidative stress, Aβ decomposition in brain tissue, neural cell death, and AD-like symptoms (McLachlan, 1986; Nie, 2018; D’Haese et al., 2019; Goher et al., 2019; Huat et al., 2019; Butterfield and Mattson, 2020; Van Dyke et al., 2020). Quercetin is known as an antioxidant with high ROS scavenger activity (Carmona-Aparicio et al., 2019; Ezequiel et al., 2019; Taïlé et al., 2020). Studies have shown that the use of QT can help to improve learning and memory, reduce ROS levels in the brain, and reduce inflammatory cytokines, apoptotic genes, and Aβ decomposition (Du et al., 2016; Li et al., 2019; Viswanatha et al., 2019; Wu et al., 2019). Therefore, QT is considered as a neuroprotective agent, but its usage has limitations because of low bioavailability (Dabeek and Marra, 2019). Accordingly, several methods have been suggested for enhancement of QT bioavailability such as non-hydrogel embedding with QT (Gallelli et al., 2020), chitosan nano-micelles conjugated with QT (Mu et al., 2019), and superparamagnetic nano-silica QT-encapsulation with PLGA (Wang et al., 2019), among which nanotechnology has been widely tried. We used aluminum to induce memory impairment in an animal model. In 2015, Lin and colleagues treated male Wistar rats with AlCl_3_ for 42 days in order to develop AD animal models and implemented behavioral tests twice on the 21st and 42nd day. Results revealed a significant increase in STL in rats treated with AlCl_3_ in comparison with the control group (Lin et al., 2015). Weng et al. (2020) demonstrated spatial memory impairment in rats treated with AlCl_3_ using behavioral tests.

The results obtained in this study revealed that AlCl_3_ treatment led to learning and memory impairment and the appearance of AD-like symptoms in rats. MWM test was used to assess changes in spatial learning and memory through escape latency, the time spent in the target zone, and the number of plate crosses. Escape latency demonstrates the time needed to find the hidden platform, and the time spent in the target zone on probe trial day shows the time that each rat spends in the quarter where the hidden platform was during training days. The number of plate crosses shows the times that each rat passes from the platform spot on the probe trial day when the platform is removed. In this study, QT-SPION treatment led to a significant decrease in the escape latency and a meaningful increase in the time spent in the target zone and the number of plates crosses in comparison with the AlCl_3_ group and showed similar results with the control group. The passive avoidance test was applied for assessment of changes in learning and memory *via* STL, which shows the time each rat needed to enter the black box on the training day and the time spent in the white box on the trial day, which shows the time that each rat stayed in the white box on the trial day. The results of the present study revealed a significant decrease in STL in the AlCl_3_ + QT-SPION group in comparison with the AlCl_3_ group while these results were similar to the results of the control group and the healthy/intact and sham group. According to the results obtained from the behavioral test, we state that treatment with QT-SPION recovers memory impairment developed by AlCl_3_ treatment. In accordance, Ebrahimpour et al. (2018) demonstrated that QT-SPION reversed memory and learning impairment induced by streptozotocin using MWM and the shuttle box.

Considering the results obtained from ICP for hippocampus tissue, Al^3+^ crosses BBB and enters brain tissue, and its accumulation causes adverse effects on hippocampal neurons such as interruption of long-term potentiation and induction of apoptosis *via* the development of oxidative stress (Gajdusek et al., 1982; Yang et al., 2004; Rather et al., 2019; Hassan and Kadry, 2020; Zhang et al., 2020). In accordance, the accumulation of Fe^3+^ in the hippocampus of SPION and QT-SPION also demonstrated that, in both groups, SPION passed BBB and entered the brain tissue. Therefore, it may also increase QT entrance into the brain tissue in the conjugate form.

AChE plays an important role in synapses and connections between neurons and muscles (Mathew et al., 2019). As AD progresses, impairments in neuron transmission also increase (Khan et al., 2018). In accordance, elevated activity of AChE is observed in the brain tissue of AD patients, and the meaningful increase in the hippocampus of rat models treated with AlCl_3_ is reported (Pourshojaei et al., 2019; Zambrano et al., 2019). This change leads to a meaningful decrease in acetylcholine levels in the brain of AD patients (Zhu et al., 2020). Accordingly, therapeutic strategies of AD that target AChE activity are highly important (Han et al., 2019; Liu et al., 2020). Several inhibitors with natural resources have been found and used to inhibit the activity of this enzyme (McHardy et al., 2017; Ansari et al., 2019; Saleem et al., 2019). In the present study, a significant increase was observed in the activity of AChE in the AlCl_3_ group. This effect was reversed in the AlCl_3_ + QT-SPION group; the progression of AD slowed down so that the levels of acetylcholine were similar to the control group. AlCl_3_ + QT showed a relative decrease in AChE activity, which was significantly lower than the AlCl_3_ group and significantly higher than the AlCl_3_ + QT-SPION and control groups. In addition, the activity level of AChE had no significant difference with the AlCl_3_ group. Therefore, it can be stated that SPION and QT cannot confront the adverse effects of AlCl_3_ effectively and AD progression is observed in the AlCl_3_ + QT and AlCl_3_ + SPION groups. Thus, the positive effects of the QT-SPION conjugate on acetylcholine levels in the hippocampus are on the basis of elevated bioavailability of QT.

Previously, it was reported that aluminum could increase the levels of reactive oxygen species in the brain tissue (Bali et al., 2019; Simunkova et al., 2019). Based on the results obtained from ICP and higher concentrations of aluminum ions, this ion crosses BBB and enters the brain tissue, while in neurons, aluminum participates in the Fenton reaction in which iron is oxidized and induces oxidative stress (Mujika et al., 2014; Yumoto et al., 2018). Currently, aluminum has been reported as an inducer of oxidative stress leading to the appearance of AD markers, so that it has been used to develop rat models of AD (Almuhayawi et al., 2020; Ogunlade et al., 2020). It is also reported that AlCl_3_-induced oxidative stress can lead to apoptosis in PC12 cells (Lu et al., 2020). In this regard, elevated oxidative stress in neurons reduces endogenous antioxidant enzymes such as catalase (CAT), glutathione peroxidase 1 (GPX), and superoxide dismutase 1 (SOD), and there was an increase in activity and levels of iNOS, which in turn produces higher levels of ROS (Guo et al., 2005; Sharma and Mishra, 2006; Sadauskiene et al., 2018; Kinawy, 2019). On the other hand, aluminum treatment develops AD hallmarks, among which increased production of Aβ is crucial (Singh et al., 2018). In the present study, qRT-PCR results of the AlCl_3_ group revealed a meaningful decrease in expression levels of antioxidant enzymes including *SOD1*, *CAT*, and *GPX1*, while *iNOS* expression levels increased significantly in comparison with the control group. In contrast, the expression levels of *SOD1*, *CAT*, and *GPX1* increased and *iNOS* expression levels decreased drastically in the AlCl_3_ + QT-SPION group, which showed the recovery effect of treatment with QT-SPION and its potential to combat the adverse effects of aluminum that helps to preserve conditions similar to the intact rats. Besides, significantly increased levels of BAX and significantly decreased expression levels of BCL2 were observed in the AlCl_3_ group in comparison with the control group, while the opposite effects were observed in AlCl_3_ + QT-SPION so that expression levels of BAX decreased and expression levels of BCL2 increased in this group in comparison with the AlCl_3_ group. According to our results, treatment with QT-SPION reduced ROS damage in neurons *via* increasing the expression levels of antioxidant enzymes and reduction of *iNOS* and increased the expression of the anti-apoptotic gene and decreased that of the pro-apoptotic gene, altogether exhibiting the beneficial effects of QT-SPION such as less neural damage and neuron survival. In addition, since the results obtained for the AlCl_3_ + QT-SPION group was closely similar to the results of the same experiments for the control group, we can state that the conjugation process elevated bioavailability of QT as much as it could protect the hippocampus against the adverse effects of aluminum and sustained conditions like the hippocampus of an intact animal.

In line with these findings, the AlCl_3_ group revealed significantly higher expression levels of the amyloid precursor protein, which is considered the most important hallmark of AD (Hamed et al., 2019; Ghammraoui and Badano, 2020). However, a significant reduction in the expression level of the amyloid precursor protein gene in the group treated with QT-SPION demonstrated its effect on inhibition of AD development and progression caused by aluminum exposure. Results obtained for mir101, the microRNA, which targets the 3′-UTR of App mRNA, showed a meaningful decrease in expression levels of this microRNA in comparison with the control group; however, treatment with the QT-SPION led to a significant increase in its expression levels in comparison with the AlCl_3_ group. Therefore, QT induced the destruction of App mRNA *via* the induction of expression of mir101. In all results obtained from the qRT-PCR experiment, QT-SPION demonstrated a significantly better performance in the induction of antioxidant genes, the anti-apoptotic gene BAX, and the Aβ antagonist mir101 compared to QT. As we mentioned before, ICP showed that SPION enters the brain, and since the AlCl_3_ + QT-SPION group showed significantly better results than the AlCl_3_ + QT and AlCl_3_ + SPION groups, then we can state that the QT-SPION conjugate crosses BBB and enters the brain and recovered adverse effects produced by aluminum.

## Conclusion

QT-SPION could be a candidate to prevent progression of AD symptoms in long-term usage. This novel compound could improve spatial learning and memory damage caused by oxidative stress in brain tissue. It also showed an inhibition effect on acetylcholinesterase and enhances expression levels of antioxidant enzymes, including catalase, glutathione peroxidase, and SOD, and a reduction in expression levels of nitric oxide synthase that helps the production of reactive oxygen species. Therefore, QT-SPION is suggested to be applied for the survival of neurons to decrease AD hallmark, App, and proapoptotic genes. The most important point to mention is that the performance of QT-SPION in all these experiments was significantly better than quercetin, which shows that the QT-SPION conjugate led to better bioavailability for quercetin and more efficient effects on the improvement of learning and memory during exposure to aluminum and AD-like conditions. There were limitations to this study, though it can be stated that the application of QT-SPION can be assessed more in order to prevent progression of AD in the early stages, which is mentioned as the main concern related to AD.

## Data Availability Statement

The datasets used and/or analyzed during the present study are available from the corresponding author upon reasonable request.

## Ethics Statement

The animal study was reviewed and approved by all procedures were conducted under the guidelines for care and use of laboratory animals (United States National Institute of Health Publication No 80-23, revised 1996) and were reviewed and approved by the animal ethics committee of the University of Isfahan.

## Author Contributions

AE conceived, designed, and supervised the study. EAJ performed the experiments and statistical analyses and interpreted the data. EAJ, SR, and MN participated in data collection and statistical analyses. All authors read and approved the final manuscript.

## Conflict of Interest

The authors declare that the research was conducted in the absence of any commercial or financial relationships that could be construed as a potential conflict of interest.
